# The predictive value of [^18^F]FDG PET/CT radiomics combined with clinical features for EGFR mutation status in different clinical staging of lung adenocarcinoma

**DOI:** 10.1186/s13550-023-00977-4

**Published:** 2023-04-04

**Authors:** Jianxiong Gao, Rong Niu, Yunmei Shi, Xiaoliang Shao, Zhenxing Jiang, Xinyu Ge, Yuetao Wang, Xiaonan Shao

**Affiliations:** 1 Department of Nuclear Medicine, The Third Affiliated Hospital of Soochow University, Changzhou, 213003 China; 2 Institute of Clinical Translation of Nuclear Medicine and Molecular Imaging, Soochow University, Changzhou, 213003 China; 3 Department of Radiology, The Third Affiliated Hospital of Soochow University, Changzhou, 213003 China; 4 Changzhou Key Laboratory of Molecular Imaging, Changzhou, 213003 China

**Keywords:** lung adenocarcinoma, [^18^F]FDG PET/CT, Radiomics, Epidermal growth factor receptor, Prediction

## Abstract

**Background:**

This study aims to construct radiomics models based on [^18^F]FDG PET/CT using multiple machine learning methods to predict the EGFR mutation status of lung adenocarcinoma and evaluate whether incorporating clinical parameters can improve the performance of radiomics models.

**Methods:**

A total of 515 patients were retrospectively collected and divided into a training set (*n* = 404) and an independent testing set (*n* = 111) according to their examination time. After semi-automatic segmentation of PET/CT images, the radiomics features were extracted, and the best feature sets of CT, PET, and PET/CT modalities were screened out. Nine radiomics models were constructed using logistic regression (LR), random forest (RF), and support vector machine (SVM) methods. According to the performance in the testing set, the best model of the three modalities was kept, and its radiomics score (Rad-score) was calculated. Furthermore, combined with the valuable clinical parameters (gender, smoking history, nodule type, CEA, SCC-Ag), a joint radiomics model was built.

**Results:**

Compared with LR and SVM, the RF Rad-score showed the best performance among the three radiomics models of CT, PET, and PET/CT (training and testing sets AUC: 0.688, 0.666, and 0.698 vs. 0.726, 0.678, and 0.704). Among the three joint models, the PET/CT joint model performed the best (training and testing sets AUC: 0.760 vs. 0.730). The further stratified analysis found that CT_RF had the best prediction effect for stage I–II lesions (training set and testing set AUC: 0.791 vs. 0.797), while PET/CT joint model had the best prediction effect for stage III–IV lesions (training and testing sets AUC: 0.722 vs. 0.723).

**Conclusions:**

Combining with clinical parameters can improve the predictive performance of PET/CT radiomics model, especially for patients with advanced lung adenocarcinoma.

**Supplementary Information:**

The online version contains supplementary material available at 10.1186/s13550-023-00977-4.

## Background

Lung cancer is the second most common cancer worldwide and has the highest mortality rate (21%) [1, 2], of which 80–85% are non-small cell lung cancer (NSCLC) [3]. Epidermal growth factor receptor (EGFR) plays an important role in the progression of NSCLC, which makes it an effective therapeutic target; tumors with EGFR mutations are more heterogeneous [4, 5]. The most common histological type of NSCLC is lung adenocarcinoma [3], which has a higher EGFR mutation rate than other subtypes [6]. In Asian patients with lung adenocarcinoma, the EGFR mutation rate is as high as 50% [7]. Studies have shown that tyrosine kinase inhibitors (TKIs) can effectively prolong the progression-free survival (PFS) of patients with EGFR mutations, and therefore they are widely used in the targeted therapy for lung adenocarcinoma [8, 9]. The therapy efficacy and prognosis are closely related to the EGFR mutation status of the patient. Accurately identifying EGFR mutation status in lung adenocarcinoma patients can greatly improve patient prognosis.

Molecular testing of needle biopsy or surgically resected tumor tissue is the "gold standard" for diagnosing EGFR mutation status. However, this technique is invasive and time-consuming, and tumor heterogeneity can easily affect its accuracy [10, 11]. In addition, many patients cannot undergo this test due to poor physical conditions and other reasons (such as fears and anxieties, concerns about potential complications, and suboptimal lesion location). In recent years, some studies used blood samples instead of biopsies to assess EGFR mutation status by analyzing circulating tumor DNA (ctDNA). However, ctDNA testing is expensive and has low sensitivity for detecting EGFR mutations [12–14]. Therefore, there is an urgent need for an economical, rapid, and reliable non-invasive detection method to assess EGFR mutation status.

[^18^F]FDG PET/CT is a non-invasive molecular imaging technique widely used in the clinical diagnosis, staging, prognosis, and efficacy evaluation of lung cancer [15–17]. The maximum standard uptake value (SUV_max_) is one of the routine parameters of PET/CT, which reflects the highest uptake value of [^18^F]FDG by the tumor tissue and is often used in PET image analysis. Radiomics is a method that quantitatively evaluates tumor imaging phenotypes via high-throughput feature extraction from medical images [18]. Compared to SUV_max_, radiomics features can better reflect the spatial distribution of tumors and more comprehensively evaluate tumor heterogeneity. In recent years, using machine learning methods with high prediction efficiency and strong feasibility to assess radiomic features and predict EGFR mutation status has become a research “hot spot” [5, 19–32]. However, most of these studies had small sample sizes, with a total sample number of no more than 200 cases [19–26], and the TNM stages of the enrolled patients varied greatly [21, 27, 31]. These factors significantly affected the stability of the results.

Studies have shown that clinical parameters such as gender, smoking history, and the presence of ground-glass opacity (GGO) are closely related to the EGFR mutation status in lung adenocarcinoma [33]. Combining with clinical parameters can improve model performance in predicting EGFR mutation status [4, 5, 26, 30], but some researchers suggest that adding clinical features to the radiomics model does not improve its predictive performance [34, 35]. Therefore, whether incorporating clinical parameters can improve the performance of the radiomics model is still inconclusive.

In this study, we included 515 patients with all clinical stages. We used LR, RF, and SVM to model CT, PET, and PET/CT radiomics features and assess the predictive power. Then, we included clinical parameters to construct joint models and evaluate whether adding clinical parameters can further improve the model performance in predicting EGFR mutation in lung adenocarcinoma patients with different clinical stages.

## Methods

### Patient data

We retrospectively and consecutively collected lung cancer patients who underwent [^18^F]FDG PET/CT examinations before treatment in our hospital from January 2018 to April 2022. Inclusion criteria: (1) Lung adenocarcinoma was confirmed by surgery or biopsy pathology, and the pathological classification was based on IASLC/ATS/ERS lung adenocarcinoma classification criteria [36]; (2) the patients completed [^18^F]FDG PET/CT examination before surgery, and the interval between surgery and examination was less than 30 days; (3) the EGFR mutation test result was available; (4) patient had no history of other malignant tumors. Exclusion criteria: (1) lesions with poor image quality or difficulty to measure; (2) patient had other subtypes of lung cancer; (3) no chest CT images.

**Fig. 1:**
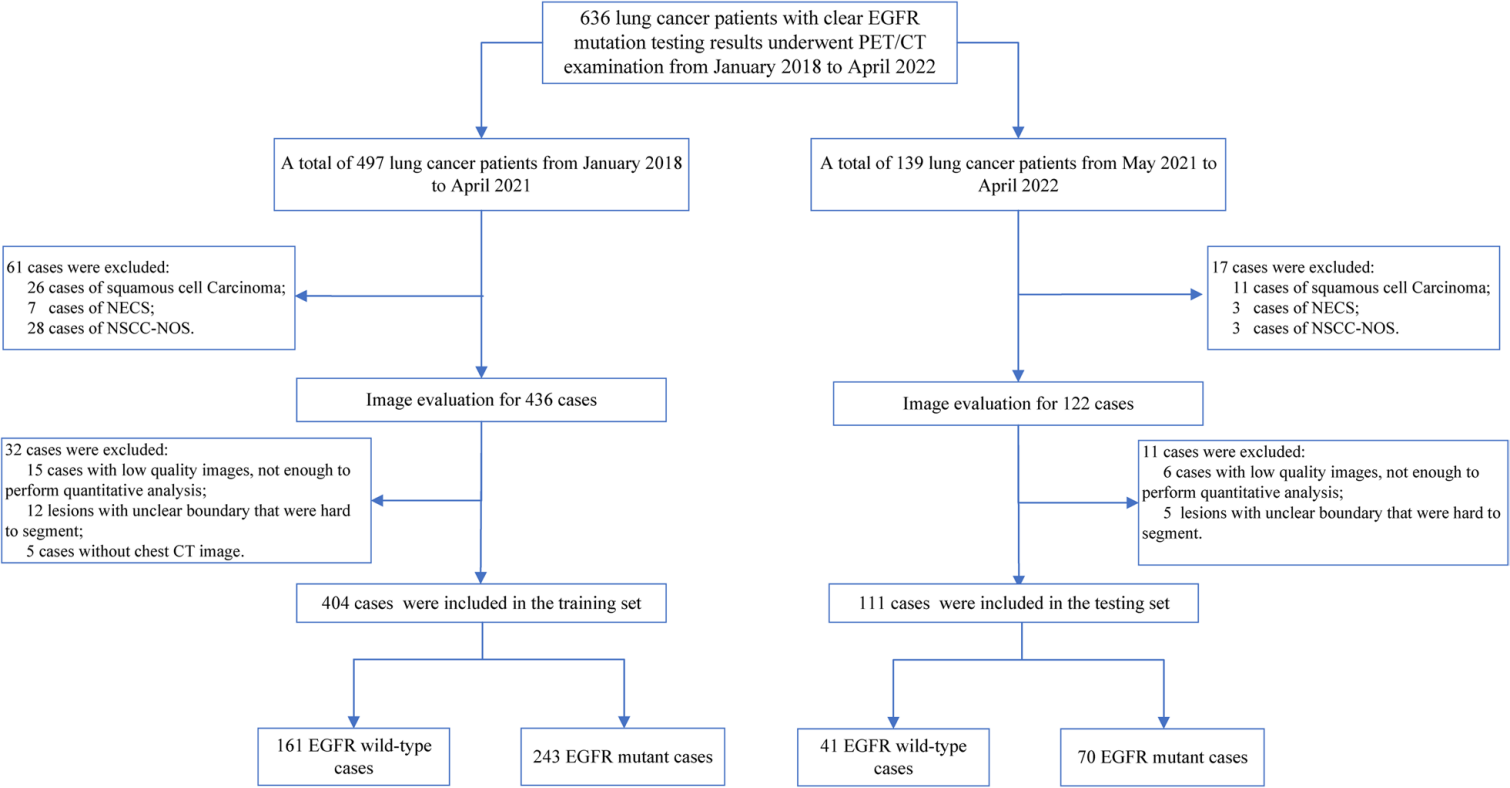
Flow chart of patient selection. *EGFR* epidermal growth factor receptor, *NECS* neuroendocrine carcinomas, *NSCC-NOS* non-small cell carcinoma-not otherwise specified

According to the above criteria, five hundred fifteen patients with lung adenocarcinoma were included, and the clinical information of age, gender, smoking status, clinical stage, tumor marker, SUV_max_, and postoperative pathology was recorded. We used patients collected from January 2018 to April 2021 as the training set and patients collected from May 2021 to April 2022 as the testing set. The study was performed in accordance with the ethical standards as laid down in the 1964 Declaration of Helsinki and its later amendments or comparable ethical standards. The study protocol was approved by the Ethics Committee of our institution (No. [2022] KD 087). As our study is a retrospective analysis, informed consent is not required. The study flow chart is shown in Fig. 1.

### EGFR mutation test

EGFR mutation test was performed on tissue specimens obtained by surgical resection or biopsy. Real-time fluorescent PCR was performed to detect EGFR mutations in exons 18–21 using the EGFR gene mutation detection kit purchased from Shanghai Yuanqi Company. The detailed procedures followed the manufacturer's instructions (see Additional file 1). If the mutation was detected in any of the above exons, the lesion was defined as EGFR mutant; otherwise, the lesion was defined as EGFR wild type.

### Image acquisition

The image acquisition protocol was developed according to the acquisition protocol based on Imaging Biomarker Standardization Initiative (IBSI) guidelines [37]. The PET/CT image acquisition instrument was a German Siemens BiographmCT (64) PET/CT machine. The patients fasted for 4–6 h before the examination, and their height, weight, and blood sugar were recorded on the examination day. [^18^F]FDG was intravenously injected according to the patient's body weight at 3.70–5.55 MBq/kg. The imaging agent was provided by Nanjing Jiangyuan Andico Positron Research and Development Company (radiochemical purity > 95%). The patients underwent PET/CT whole-body imaging after resting in a quiet and comfortable environment for 1 h. The patient was placed in the supine position and kept holding the head with both hands. Imaging lasted for 2 min/bed, and the collection range was from the base of the skull to the middle of the femur. Diagnostic chest CT imaging was performed after the PET/CT scan. After image acquisition, the TrueX + TOF (ultraHD-PET) system was used for image reconstruction. A postprocessing workstation TrueD system (Siemens) was used for image evaluation. Image acquisition parameters are listed in Additional file.

### Image analysis and tumor region segmentation

[^18^F]FDG PET/CT image analysis: PET and CT images were analyzed by two physicians (A and B) with 3 years of experience in nuclear medicine. The software they used was the 3D slicer software 4.11.2 (http://www.slicer.org). For PET images, they used a semi-automatic segmentation method developed by Beichel et al. [38]. For CT images (3 mm), they used NVIDIA AI-Assisted Annotation (3D-Slicer built-in) and the boundary-based CT segmentation models to process lung nodule images. The segmented tumor region of interest (ROI) was checked and proofread by another physician with more than 10 years of experience in PET/CT diagnosis. Four weeks after completing the ROI for all cases, Doctor A segmented the tumor region again for 300 patients, among which 70 patients were randomly selected for Doctor B to segment.

### Image preprocessing

Before feature extraction, the image was normalized, and all images were interpolated (sitkBSpline algorithm, 3rd-order B-spline interpolation) so that the isotropic voxel spacing was rotated unchanged and the extracted features were compared between different samples. CT images were resampled to 1 × 1 × 1 mm^3^, and PET images were resampled to 3 × 3 × 3 mm^3^. The method of fixed bin width was used for discretization, and the bin width of CT and PET images was 25 and 0.313, respectively. The bin discretization, Laplacian of Gaussian (LOG), and wavelet transform were applied to generate different feature sets. For the LOG filter, different sigma values were used to extract fine, medium, and coarse features; specifically, these features ranged from 0.5 to 5 with a step size of 0.5. The wavelet transforms produced 8 decompositions per layer (applying all possible combinations of high-pass or low-pass filters in each of the three dimensions, including HHH, HHL, HLH, HLL, LHH, LHL, LLH, and LLL). All intensity, histogram, and texture features were preprocessed (including discretization, logarithm, and wavelet).

### Feature extraction

Using the Pyradiomics module in Python 3.8.8, we extracted multiple features from different feature categories based on three segmented ROIs (twice from Doctor A and once from Doctor B). These categories included shape and morphology-based features (14 shape features), first-order statistics (18 FOS features), gray-level co-occurrence matrix (GLCM 24 features), gray-level dependency matrix (14 GLDM features), gray-level run-length matrix (16 GLRLM features), gray-level size region matrix (16 GLSZM features), and the neighbor gray-level tone difference matrix (5 NGTDM features). The features extracted from three sets of ROIs were assessed for within-group and between-group intraclass correlation efficient (ICC), and the features with ICCs greater than 0.75 were considered in good consistency and kept for further analysis.

### Screening of radiomic features and model construction

To avoid overfitting, the variance method was used to remove features with small variance (threshold = 0.24). Next, the Mann–Whitney *U* test was used in the training set to screen out radiomics features with *p* value < 0.1, which might be associated with EGFR mutation status. Then, the Least Absolute Shrinkage and Selection Operator (LASSO) algorithm was applied to the normalized training set data to select the best predictive features. The LASSO algorithm added an L1 regularization term to the least squares algorithm to avoid overfitting and employed fivefold cross-validation.

**Fig. 2:**
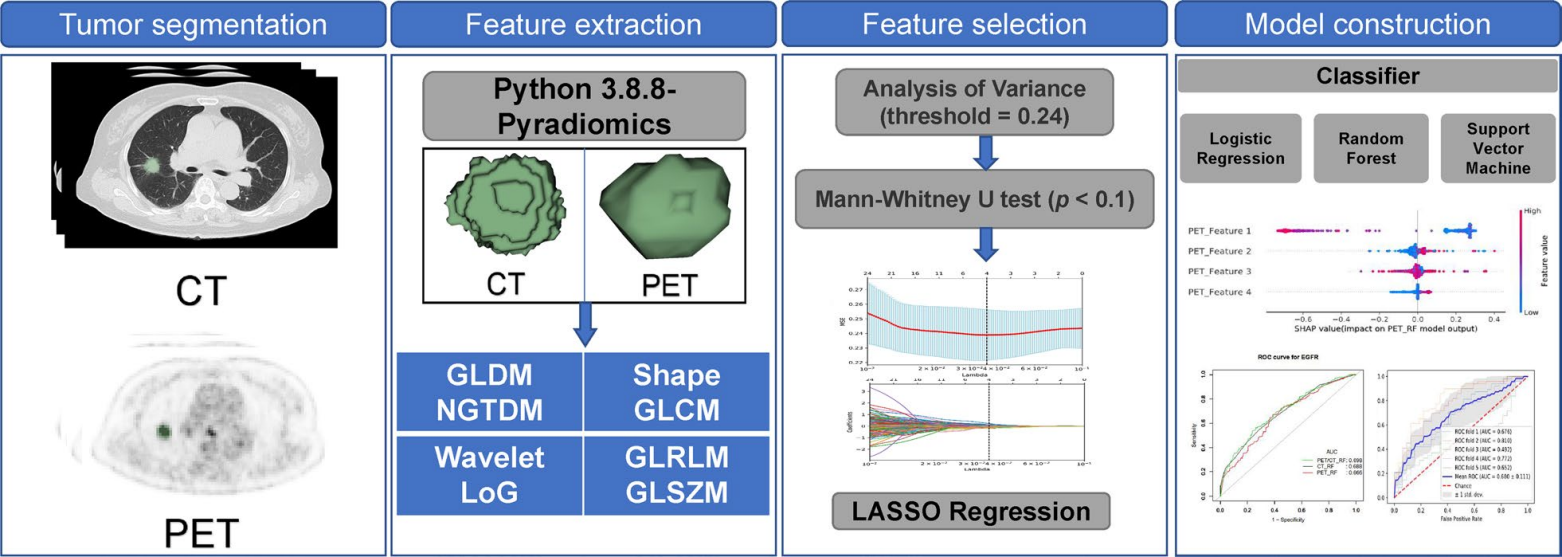
Flow chart of radiomics analysis

Machine learning models were built using the Sklearn module in Python 3.8.8. Nine models were constructed based on CT, PET, and PET/CT radiomics features with LR, SVM, and RF, respectively. The training set used a grid search with fivefold cross-validation to find the optimal hyperparameters (the specific parameters are listed in Additional file 1: Table S1), and the models were retrained on the entire training set. Receiver operating characteristic (ROC) curves and the area under the curve (AUC) were used to evaluate model performance in training and testing sets. The 3 models with the best performance on the testing set were kept to calculate the Rad-score. We used the SHAP module in Python 3.8.8 to interpret the model better to understand the importance of different features in different models. The biggest advantage of the SHAP value is that SHAP can reflect the positive and negative influence of the features in each sample. The radiomics workflow used in this study is shown in Fig. 2.

### Statistical analysis

Statistical analysis was performed using R software (version 3.4.3; http://www.R-project.org/). Continuous variables were expressed as mean ± standard deviation (normal distribution) or median (Q1–Q3) (skewed distribution). Categorical variables were expressed as frequency or rate (%). Differences in clinical data and PET/CT metabolic parameters between different EGFR mutation status (binary variables) were tested using the χ^2^ test (for categorical variables), *T* test (for normal distribution), or Mann–Whitney *U* test (skewed distribution). Multivariate logistic regression method was used to construct a clinical model with significant clinical parameters, and a joint model and the corresponding nomogram were built by combining the clinical parameters with three Rad-scores. The minimum Akaike information criterion was used to select the best model parameters. The calibration curve confirmed the agreement between the nomogram and observations, and the model’s power was evaluated using ROC curve and AUC. The model's accuracy, sensitivity, and specificity were calculated to obtain a quantitative performance measurement. Decision curve analysis (DCA) assessed the models' clinical utility and net clinical benefit. A pairwise comparison of the model AUCs was performed using the method proposed by Delong et al. [39]. All statistical tests were two-sided, and *p* < 0.05 was considered statistically significant. Since the carcinoembryonic antigen (CEA) of 49 cases (9.5%), the cytokeratin 19 fragment (CYFRA21-1) of 99 cases (19.1%), the neuron-specific enolase (NSE) of 74 cases (14.3%), the serum squamous cell carcinoma antigen (SCC-Ag) of 91 cases (17.6%) were missing, we imputed the missing data using miceforest (version 5.4.0; https://github.com/AnotherSamWilson/miceforest/).

## Results

### Clinical characteristics of patients

The clinical characteristics of the patients are shown in Table 1. A total of 515 patients with lung adenocarcinoma were enrolled in the study, including 264 females and 251 males. The average age was 64.0 ± 9.2 years (ranging from 36 to 87 years). In total, 175 patients (34.0%) had a smoking history, and 348 (67.6%) had solid nodules. The patients’ clinical stages were: stage I: 209 cases (40.6%), stage II: 24 cases (4.7%), stage III: 85 cases (16.5%), and stage IV: 197 cases (38.3%). The EGFR mutation status was pathologically confirmed by surgical resection or biopsy: there were 202 cases (39.2%) with EGFR wild type and 313 cases (60.8%) with EGFR mutant (including 3 cases of exon 18, 127 cases of exon 19, 13 cases of exon 20, 150 cases of exon 21, 1 case of exon 19 + 20, 2 cases of exon 19 + 21, 1 case of exon 20 + 21, and 16 cases of the unknown exon).

**Table 1:** Clinical characteristics of patients with different EGFR mutation status in training set and testing set

EGFR	Training set *n* = 404	Mutant	*p* value	Testing set n = 111	Mutant	*p* value
	Wild type			Wild type		
N	161	243		41	70	
Age (years)	64.83 (9.11)	63.65 (9.22)	0.210	64.22 (8.75)	63.19 (9.48)	0.570
Gender			< 0.001			0.021
Female	51 (31.7%)	162 (66.7%)		13 (31.7%)	38 (54.3%)	
Male	110 (68.3%)	81 (33.3%)		28 (68.3%)	32 (45.7%)	
Smoking history	87 (54.0%)	54 (22.2%)	< 0.001	18 (43.9%)	16 (22.9%)	0.020
Nodule type			< 0.001			0.037
Solidity	128 (79.5%)	147 (60.5%)		32 (78.0%)	41 (57.6%)	
Sub-solidity	33 (20.5%)	96 (39.5%)		9 (22.0%)	29 (42.4%)	
Nodule location			0.645			0.696
Top right	47 (29.2%)	81 (33.3%)		10 (24.4%)	21 (30.0%)	
Middle right	6 (3.7%)	14 (5.8%)		4 (9.8%)	8 (11.4%)	
Lower right	34 (21.2%)	50 (20.6%)		12 (29.2%)	14 (20.0%)	
Top left	44 (27.3%)	63 (25.9%)		9 (22.0%)	20 (28.6%)	
Lower left	30 (18.6%)	35 (14.4%)		6 (14.6%)	7 (10.0%)	
Tumor long axis (mm)	32.10 (20.70–47.20)	25.60 (20.30–37.65)	0.002	40.70 (29.50–49.50)	29.40 (23.70–38.60)	0.008
Tumor short axis (mm)	23.20 (14.60–33.20)	19.00 (14.30–27.65)	0.015	29.60 (24.10–33.40)	22.35 (16.20–28.45)	0.008
Clinical stage			0.005			0.378
I	52 (32.3%)	125 (51.4%)		8 (19.5%)	24 (34.3%)	
II	12 (7.5%)	3 (1.2%)		4 (9.8%)	5 (7.1%)	
III	33 (20.5%)	35 (14.4%)		8 (19.5%)	9 (12.9%)	
IV	64 (39.8%)	80 (32.9%)		21 (51.2%)	32 (45.7%)	
CEA (ng/ml)	5.24 (2.61–15.61)	3.23 (1.60–12.25)	0.016	5.08 (2.42–13.39)	5.58 (2.24–19.84)	0.781
CYFRA 21–1 (ng/ml)	3.58 (2.42–6.20)	3.23 (2.17–5.34)	0.084	4.36 (3.24–6.43)	3.73 (2.58–6.90)	0.313
NSE (ng/ml)	15.10 (11.79–20.28)	14.75 (12.04–20.38)	0.988	15.81 (13.07–20.55)	14.97 (12.14–18.64)	0.281
SCC-Ag (ng/ml)	1.01 (0.72–1.51)	0.78 (0.56–1.01)	< 0.001	0.89 (0.72–1.29)	0.83 (0.56–1.18)	0.350
SUV_max_ (ng/ml)	13.03(6.27–18.21)	10.14(3.44–17.51)	0.005	15.60(8.51–20.67)	13.62(5.50–17.98)	0.139

Mean (SD)/Median (Q1–Q3)/N (%).

*EGFR* epidermal growth factor receptor; *CEA* carcinoembryonic antigen; *CYFRA 21–1* cytokeratin 19 fragment; *NSE* neuron-specific enolase; *SCC-Ag* squamous cell carcinoma-associated antigen; *SUV*_*max*_ maximum standardized uptake value

There were no significant differences between the training set (*n* = 404) and testing set (*n* = 111) in age, gender, smoking history, nodule type, nodule location, tumor markers, and EGFR mutation rate (all *p* > 0.05). Tumor long axis, tumor short axis, clinical stage, and SUV_max_ showed significant differences between the two datasets (all *p* < 0.05), possibly due to the different compositions of patients during different periods (see Additional file 1: Table S2). To eliminate this difference, we performed a stratified analysis (stratified by clinical stage) in both datasets to verify the robustness of the joint model. The clinical parameters of gender, smoking history, nodule type, CEA, SCC-Ag, clinical stage, tumor long axis, and tumor short axis explained the differences between EGFR mutant and wild-type patients (all *p* < 0.05 in training set).

### Validation of the predictive efficacy of traditional metabolic parameters

SUV_max_ was significantly different between EGFR mutant and wild type in the training set (*p* = 0.005) but not in the testing set (*p* > 0.05). SUV_max_ had a weak predictive ability for EGFR mutation status in lung adenocarcinoma (training set AUC = 0.582, 95% CI 0.526–0.638, testing set AUC = 0.584, 95% C0I 0.475–0.694).

### The screening results of the three modality radiomics features

Based on the segmentations of tumor regions on PET/CT images, a total of 3562 radiomics features (1781 PET features, 1781 CT features) were extracted. Among them, 423 CT features and 248 PET features were excluded based on intragroup ICCs evaluation, and 109 CT features and 81 PET features were excluded according to intergroup ICCs evaluation. Next, we used the variance method, Mann–Whitney *U* test, and LASSO algorithm (Additional file 1:Figure S1) to further screen out 8 CT features, 4 PET features, and 4 PET/CT fusion radiomics features (2CT + 2PET), respectively (Additional file 1:Table S3).

### The predictive power of the three modality radiomics features for EGFR mutation status

**Fig. 3:**
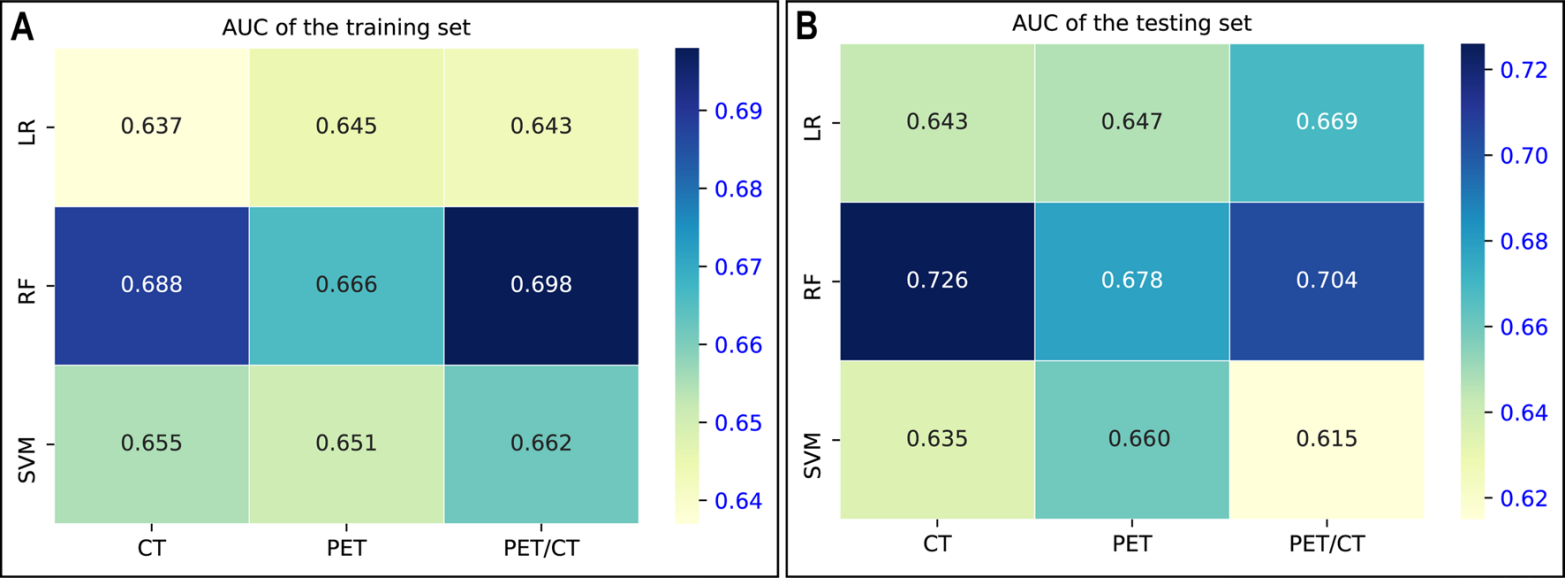
The AUC values of the nine radiomics models in the training set (**A**) and testing set **B**. *LR* logistic regression, *RF* random forest, *SVM* support vector machine

The corresponding AUCs of the radiomics models are shown in Fig. 3, and the feature weights for each model are shown in Additional file 1:Figure S2. Among the 9 constructed radiomics models, the three models based on RF algorithm were better than the models based on LR and SVM algorithms in both training set and testing set, and the AUCs of CT_RF, PET_RF, and PET/CT_RF in the training set were 0.688, 0.666, and 0.698, respectively, and the AUCs in the testing set were 0.726, 0.678, and 0.704, respectively. Although the performance of CT_RF model was better than that of PET_RF and PET/CT_RF models in the testing set, the difference was not significant (both *p* > 0.05). Table 2 lists the diagnostic efficacy of the three best models.

**Table 2:** Comparison of the predictive performance of clinical model, radiomics models, and joint models

	AUC	95% CI low	95% CI up	Best threshold	Specificity	Sensitivity	Accuracy
*Training set*							
Clinical model	0.738	0.688	0.788	0.643	0.683	0.708	0.698
CT_RF	0.688	0.637	0.739	0.635	0.714	0.547	0.614
CT joint model	0.773	0.727	0.818	0.638	0.795	0.638	0.701
PET_RF	0.666	0.613	0.719	0.525	0.615	0.670	0.649
PET joint model	0.743	0.694	0.792	0.533	0.590	0.794	0.713
PET/CT_RF	0.698	0.647	0.749	0.549	0.739	0.564	0.634
PET/CT joint model	0.760	0.713	0.807	0.573	0.683	0.733	0.713
*Testing set*							
Clinical model	0.681	0.577	0.782	0.538	0.634	0.686	0.667
CT_RF	0.726	0.629	0.822	0.596	0.756	0.643	0.685
CT joint model	0.723	0.628	0.818	0.504	0.683	0.671	0.676
PET_RF	0.678	0.572	0.785	0.504	0.659	0.643	0.649
PET joint model	0.703	0.601	0.806	0.575	0.634	0.686	0.667
PET/CT_RF	0.704	0.603	0.804	0.426	0.561	0.800	0.712
PET/CT joint model	0.730	0.633	0.828	0.491	0.585	0.786	0.712

*AUC* area under the curve; *CI* confidence interval; *RF* random forest

We further compared the performance of CT_RF, PET_RF, PET/CT_RF, and SUV_max_ for predicting EGFR mutation status. The ROC curves of the three radiomics models and SUV_max_ in training set and testing set are shown in Additional file 1: Figure S3. The AUCs of three radiomics models were significantly better than SUV_max_ in both training and testing sets (all *p* < 0.05).

### The performance of radiomics model combined with clinical parameters for predicting EGFR mutation status

We first constructed a clinical prediction model (baseline model) with the 8 clinical parameters in Table 1 that might be related to the EGFR mutation status. Five parameters were finally included, and the model equation is as follows:

Logit(P) = 0.91617 + 0.00156 × CEA − 0.05743 × SCC-Ag − 0.92507 × (Gender = male) − 0.57848 × (Smoking history = positive) + 0.70786 × (Nodule Type = sub-solidity).

Next, the above 5 parameters were combined with the three best radiomics models (CT_RF, PET_RF, and PET/CT_RF) to construct joint prediction models. The three joint models are as follows:

CT joint model:

Logit(P) = − 2.11428 + 0.00177 × CEA − 1.31985 × (Gender = male) + 0.52447 × (Nodule Type = sub-solidity) + 4.96787 × CT_Rad;

PET joint model:

Logit(P) = − 1.01180 + 0.00170 × CEA –0.86919 × (Gender = male)− 0.58352 × (Smoking history = positive) + 4.02651 × PET_Rad;

PET/CT joint model:

Logit(P) = − 1.66386 + 0.00183 × CEA –0.89610 × (Gender = male) − 0.53267 × (Smoking.history = positive) + 5.20912 × PET/CT_Rad.

**Fig. 4:**
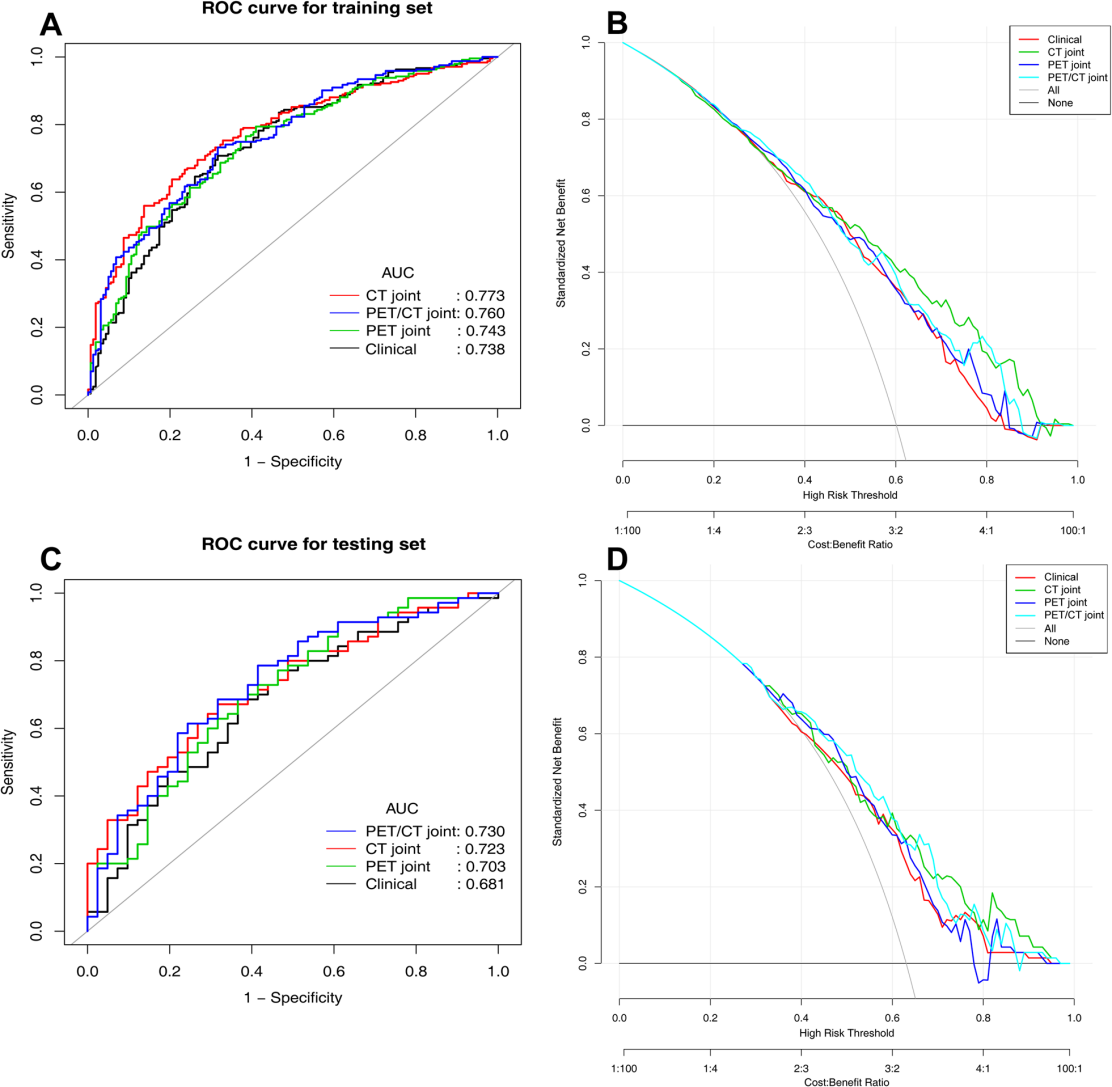
ROC and DCA curves of the clinical model and CT, PET, PET/CT joint model in the training set (**A**–**B**) and testing set (**C**–**D**). *ROC* receiver operating characteristic, *AUC* area under the curve

The ROC and DCA curves of the clinical and three joint models are shown in Fig. 4, and the diagnostic efficacy is shown in Table 2. In the training set, CT_joint model had the highest specificity of 0.795, while the PET joint model and the PET/CT joint model had the highest accuracy of 0.713, and the PET joint model had the highest sensitivity of 0.794. In the testing set, CT_RF had the highest specificity of 0.756, while the PET/CT_RF and the PET/CT joint model had the highest accuracy of 0.712, and the PET/CT_RF model had the highest sensitivity of 0.800.

The AUCs of three joint models and clinical model in the training set followed the order of CT joint model > PET/CT joint model > PET joint model > clinical model, but only the AUC of CT joint model was significantly better than the clinical model (*p* = 0.049). The AUC values in the testing set followed the order of PET/CT joint model > CT joint model > PET joint model > clinical model, but the differences were not significant (*p* > 0.05). By calculating the net reclassification index (NRI), we found that the PET/CT joint model correctly reclassified 10.0% (95% CI 1.0–19.0%, *p* = 0.029) of mutant type more than the clinical model, while there was no significant difference in identifying wild type (*p* = 0.410). Further comparison of DCA showed that the net benefit of the three joint models was higher than that of the clinical model in both training and testing sets (Fig. 4).

**Fig. 5:**
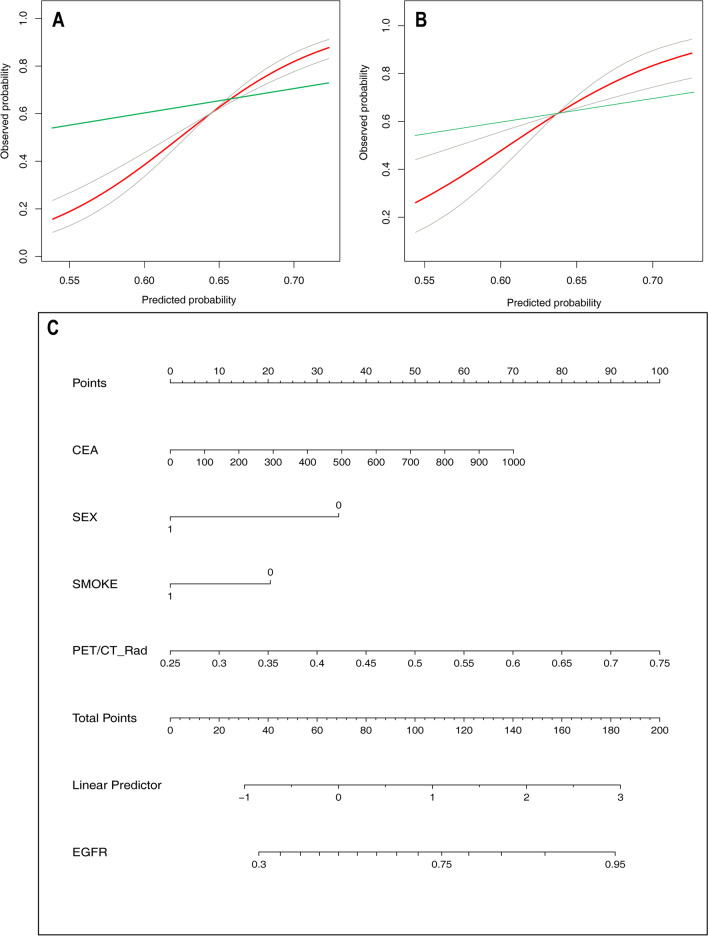
The nomogram and calibration curves of the PET/CT joint model for predicting EGFR mutation in lung adenocarcinoma. **A** The calibration curve in the training set. **B** The calibration curve in the testing set. **C** The nomogram. The horizontal axis is the predicted incidence of the EGFR mutation, and the vertical axis is the observed incidence of the EGFR mutation. The red line is the reference line, indicating that the predicted value is equal to the actual value. The green line is the calibration curve, and the areas between two gray line represent the 95% CI on both sides

We further performed stratified analysis to verify the diagnostic performance of radiomics models, clinical models, and joint models in different clinical stage (Table 3). In the training set, clinical stage significantly affected the prediction of EGFR mutation status by the three radiomics models and the joint PET/CT model, suggesting there was an interaction effect (all *p* < 0.05); however, this effect was not significant in the testing set (*p* = 0.067–0.869). The EGFR mutation rates and clinical characteristics of the patients with different clinical stages are shown in Additional file 1: Table S4. For stage I-II nodules, besides clinical model, other models all showed varying degrees of overfitting. The CT joint model performed best in the training set (AUC: 0.838), while the CT_RF model performed the best in the testing set (AUC: 0.797). For stage III-IV nodules, the clinical model performed the best in the training set (AUC: 0.729), followed by the PET/CT joint model (AUC: 0.722); in the testing set, the clinical model showed significant overfitting (AUC: 0.675), while the PET/CT joint model showed the best performance (AUC: 0.723). The nomogram and a calibration curve of the PET/CT joint model are shown in Fig. 5.

**Table 3:** Predictive performance of radiomics models, clinical model, and three joint models in different clinical stages

Model	Stage I–II *n* = 233	Stage III–IV *n* = 282	p for interaction
	AUC (95% CI)	AUC (95% CI)	
*Training set*			
Clinical model	0.728 (0.650–0.806)	0.729 (0.661–0.797)	0.856
CT_RF	0.791 (0.727–0.856)	0.576 (0.499–0.653)	< 0.001
CT joint model	0.838 (0.779–0.896)	0.699 (0.628–0.769)	0.001
PET_RF	0.711 (0.632–0.789)	0.589 (0.511–0.666)	0.005
PET joint model	0.753 (0.680–0.826)	0.720 (0.652–0.788)	0.251
PET/CT_RF	0.802 (0.737–0.867)	0.612 (0.534–0.689)	< 0.001
PET/CT joint model	0.794 (0.727–0.860)	0.722 (0.653–0.790)	0.032
*Testing set*			
Clinical model	0.655 (0.446–0.865)	0.675 (0.547–0.804)	0.810
CT_RF	0.797 (0.634–0.961)	0.665 (0.537–0.794)	0.067
CT joint model	0.782 (0.634–0.929)	0.682 (0.556–0.808)	0.520
PET_RF	0.625 (0.439–0.811)	0.707 (0.577–0.836)	0.316
PET joint model	0.675 (0.481–0.870)	0.707 (0.580–0.835)	0.869
PET/CT_RF	0.744 (0.582–0.961)	0.702 (0.573–0.830)	0.784
PET/CT joint model	0.750 (0.589–0.911)	0.723 (0.599–0.846)	0.837

*AUC* area under the curve; *CI* confidence interval; *RF* random forest

## Discussion

This article aims to use machine learning methods to construct a radiomics model based on ^18^F-FDG PET/CT images to predict the EGFR mutation status in lung adenocarcinoma patients and evaluate the added value of clinical parameters in improving the predictive performance of the radiomics model. CT_RF, PET_RF, and PET/CT_RF achieved moderate predictive performance (training and testing sets AUC: 0.688, 0.666, and 0.698 vs. 0.726, 0.678, and 0.704, respectively). Furthermore, the PET/CT joint model had the best predictive performance (training and testing sets AUCs: 0.760 vs. 0.730), especially in the advanced lung adenocarcinoma subgroup (training and testing sets AUCs: 0.722 vs. 0.723).

Recent meta-analysis confirmed that SUV_max_ had moderate predictive power for EGFR mutation status (AUC: 0.68–0.69) [40, 41]. Our study found that the predictive ability of SUV_max_ for EGFR mutation status was weak (AUC: 0.584), possibly because our study only included lung adenocarcinomas. In this study, three radiomics models based on RF were finally selected, and their prediction performance was all significantly better than SUV_max_, which is consistent with the results from Zhang et al. [42]. The possible reason is that radiomics features can better reflect the spatial distribution of tumors than the traditional metabolic parameters, thereby more comprehensively evaluating the tumor heterogeneity. For the specific radiomics features in the model, the one with the highest weight in CT_RF was original_firstorder_Median, which represents the median grayscale intensity of the CT image, indicating that the lower the nodule density, the higher the probability of EGFR mutation. original_shape_Maximum2DdiameterColumn was the feature with the highest weight in the PET_RF model. It represents the nodule length, and a smaller length is associated with a higher EGFR mutation rate. In the PET/CT_RF model, original_firstorder_Media and original_shape_Maximum2DdiameterColumn were still the two radiomics features with the highest weights, which confirmed the robustness of these features.

The clinical characteristics of lung adenocarcinoma patients are important variables in evaluating EGFR mutation status. We found that the lesion size and clinical stage of the EGFR mutation group were significantly lower than those of the wild-type group, suggesting that the lesions in mutation group were smaller and earlier in stage. Moreover, the subsolid nodules had a higher EGFR mutation rate, which is consistent with other reports [33, 43]. Several other studies have shown that female gender and no smoking history are associated with EGFR mutations [8, 44, 45], which is supported by our findings. In this study, the clinical model had a certain predictive value for EGFR mutation status (AUC: 0.681), of which CEA level and gender were kept in the subsequent joint models, and higher CEA level and female gender were independent predictors for EGFR mutation. In the testing set, the predictive performance of PET joint model and PET/CT joint model was further improved compared to the original radiomics models, which is consistent with previous studies [4, 29].

Lung adenocarcinoma patients with different clinical stages have different treatment options. Patients with advanced stages are often inoperable, and the treatment relies more on traditional chemotherapy and targeted therapy. In this study, we found that the CT_RF model had the best prediction power on stage I–II lesions, and the combination with clinical parameters did not improve its predictive performance, which might be related to the high proportion of subsolid nodules in stage I–II nodules. Yang et al. [34] and Cheng et al. [35] obtained similar results in subsolid nodules. For stage III–IV nodules, the PET/CT joint model was the best at predicting EGFR mutation status, and it was better than PET/CT_RF. Since stage III–IV patients are more dependent on targeted therapy, the PET/CT joint model can assist clinicians in making more precise treatment decisions for advanced patients.

Compared to previous studies, a major advantage of our research is that we had a large sample size and included all stages, and through stratified analysis by clinical stage, it provided the optimal population for the radiomics model in clinical practice. There are still some limitations of this study. First, this study is a single-center retrospective study with fewer patients in stage II; thus, the model needs to be further verified prospectively in external datasets. Secondly, Beig et al. [46] believed that the CT radiomics features of the surrounding area of nodules also have certain predictive values, but we did not include them when performing the segmentation; thus, we might lose some information around the tumor, and further research is needed to determine the optimal parameter values for image reconstruction and preprocessing. Third, the stratified analysis showed that the clinical stage impacted the model’s prediction performance, and it is necessary to build a separate model for early lung adenocarcinoma. Fourth, we did not include CT semantic features (such as burrs, vacuoles, lobulation) in the study; although some studies believe that these features are related to EGFR mutation status [47, 48], the process of semantic feature labeling is highly observer-dependent, with significant inter-observer variability [45, 49, 50]. Fifth, to include more samples in the study, we did not use 1-mm CT images, which could affect the performance of CT radiomics model [51].

## Conclusion

The [^18^F]FDG PET/CT radiomics models constructed using machine learning algorithms were a potential non-invasive method to identify EGFR mutation status in patients with lung adenocarcinoma. The clinical stage could affect the model’s prediction performance, and the PET/CT joint model was more effective in predicting the EGFR mutation status in patients with advanced lung adenocarcinoma. The different models based on PET/CT radiomics features and clinical parameters can help guide clinical decision-making and promote individualized and precise targeted therapy for patients in different clinical stages.

## Supplementary Information

**Additional file 1**.** Table S1**. List of best parameter configurations for 9 machine learning algorithm.** Table S2**. Clinical characteristics and the EGFR mutation rate of patients in training set and testing set.** Table S3**. Radiomics features used by the three modality models.** Table S4**. Clinical characteristics and the EGFR mutation rate of patients in clinical stage I-II group and III-IV group.** Figure S1**. The LASSO algorithm and 5-fold cross-validation were used to extract the optimal subset of radiomics features.** Figure S2**. SHAP value graph of CT, PET and PET/CT radiomics models.** Figure S3**. The ROC curve of the three best radiomics models and SUVmax for identifying EGFR mutation status in training set and testing set.

## Data Availability

All data generated or analyzed during this study are available from the corresponding author Xiaonan Shao upon reasonable request.

## Abbreviations

NSCLC: Non-small cell lung cancer
ctDNA: Circulating tumor DNA
IBSI: Imaging Biomarker Standardization Initiative
ROI: Region of interest
LOG: Laplacian of Gaussian
LASSO: Least Absolute Shrinkage and Selection Operator
AUC: Area under the curve
ROC: Receiver operating characteristic
DCA: Decision curve analysis
CEA: Carcinoembryonic antigen
SCC-Ag: Squamous cell carcinoma antigen
EGFR: Epidermal growth factor receptor
CYFRA 21–1: Cytokeratin 19 fragment
NSE: Neuron-specific enolase
SUV_max_: Maximum standardized uptake value
ICC: Intraclass correlation efficient
LR: Logistic regression
RF: Random forest
SVM: Support vector machine
NRI: Net reclassification index
Rad-score: Radiomics score
TKIs: Tyrosine kinase inhibitors
PFS: Prolong the progression-free survival
GGO: Ground-glass opacity
