# Supplementary material for: The predictive value of [18F]FDG PET/CT radiomics combined with clinical features for EGFR mutation status in different clinical staging of lung adenocarcinoma
Source: EJNMMI Res. 2023 Apr 4;13:26. doi: 10.1186/s13550-023-00977-4 (PMC10073367; doi:10.1186/s13550-023-00977-4)
Supplement: Supplementary file 1 — Additional file 1. Table S1. List of best parameter configurations for 9 machine learning algorithm. Table S2. Clinical characteristics and the EGFR mutation rate of patients in training set and testing set. Table S3. Radiomics features used by the three modality models. Table S4. Clinical characteristics and the EGFR mutation rate of patients in clinical stage I-II group and III-IV group. Figure S1. The LASSO algorithm and 5-fold cross-validation were used to extract the optimal subset of radiomics features. Figure S2. SHAP value graph of CT, PET and PET/CT radiomics models. Figure S3. The ROC curve of the three best radiomics models and SUVmax for identifying EGFR mutation status in training set and testing set. [file 13550_2023_977_MOESM1_ESM.docx]

**Supplementary material**

**EGFR Detection Methods**

The DNA was extracted from samples such as paraffin-embedded pathological tissues or sections of patients and amplified on ABI 7300 fluorescence PCR detector. The amplification conditions were: 42℃, 5min; 94℃, 3min; (94℃, 15sec; 60℃, 60sec) for 40 cycles; the reaction volume was 25µL; the fluorescence signal was collected at 60℃ in the second step of the PCR cycle; the detection channel was FAM-TAMRA, and the reference fluorescence was set to none. The computer automatically processed and analyzed the data.

**Image acquisition parameters**

Scanning and reconstruction parameters of diagnostic chest CT imaging: tube voltage 140 kV, tube current was automatically adjusted by caredose software according to human anatomy and tissue density, rotation time 0.5 s/turn, pitch 0.6, slice thickness 3.0 mm, matrix 512 × 512, lung window (window width 1200 HU, window level -600 HU), mediastinal window (window width 350 HU, window level 40 HU). Image reconstruction was performed according to the slice thickness of 3.0 mm.

**Table S1: List of best parameter configurations for 9 machine learning algorithms**

| **Model** | **Parameter configuration** |
| --- | --- |
| **CT_LR** | penalty="l1"  solver="liblinear"  max_iter=1000  class_weight="balanced" |
| **CT_RF** | class_weight = 'balanced'  n_estimators = 26  bootstrap = True  max_depth = 8  max_samples = 24 |
| **CT_SVM** | C=0.0354248246610472  class_weight='balanced'  gamma='auto', kernel='poly'  probability=True  'kernel': 'rbf' |
| **PET_LR** | penalty="l1"  solver="liblinear"  max_iter=1000  class_weight="balanced" |
| **PET_RF** | class_weight = 'balanced'  n_estimators = 63  bootstrap = True  max_depth = 3  min_impurity_decrease = 0.02  max_samples = 403 |
| **PET_SVM** | C=1.80155941358639  class_weight='balanced'  gamma='auto',  probability=True  'kernel': 'rbf' |
| **PET/CT_LR** | penalty="l1"  solver="liblinear"  max_iter=1000  class_weight="balanced" |
| **PET/CT_RF** | class_weight = 'balanced'  n_estimators = 15  bootstrap = True  max_depth = 2  min_samples_leaf = 7  min_samples_split = 0.07  max_samples = 165 |
| **PET/CT_SVM** | C=0.21824493848313825  class_weight='balanced'  gamma='auto'  kernel='poly'  probability=True |

**Note:** LR = logistic regression, RF = random forest, SVM = support vector machine.

**Table S2: Clinical characteristics and the EGFR mutation rate of patients in training set and testing set**

| GROUP | Training set | Testing set | P-value |
| --- | --- | --- | --- |
| N | 404 | 111 |  |
| Age (years) | 64.12 (9.18) | 63.54 (9.15) | 0.574 |
| Gender |  |  | 0.206 |
| Female | 213 (52.72%) | 51 (45.95%) |  |
| Male | 191 (47.28%) | 60 (54.05%) |  |
| Smoking history | 141 (34.90%) | 34 (30.63%) | 0.400 |
| Nodule type |  |  | 0.646 |
| Solidity | 275 (68.07%) | 73 (65.77%) |  |
| Sub-solidity | 129 (31.93%) | 38 (34.23%) |  |
| Nodule location |  |  | 0.161 |
| Top right | 128 (31.68%) | 31 (27.93%) |  |
| Middle right | 20 (4.95%) | 12 (10.81%) |  |
| Lower right | 84 (20.79%) | 26 (23.42%) |  |
| Top left | 107 (26.49%) | 29 (26.13%) |  |
| Lower left | 65 (16.09%) | 13 (11.71%) |  |
| Tumor long axis | 27.00 (20.40-40.52) | 33.20 (24.80-45.90) | 0.006 |
| Tumor short axis (mm) | 19.70 (14.57-29.75) | 25.40 (17.15-31.05) | 0.010 |
| Clinical stage |  |  | 0.008 |
| I | 177 (43.81%) | 32 (28.83%) |  |
| II | 15 (3.71%) | 9 (8.11%) |  |
| III | 68 (16.83%) | 17 (15.32%) |  |
| IV | 144 (35.64%) | 53 (47.75%) |  |
| CEA (ng/ml) | 4.08 (1.90-13.00) | 5.28 (2.38-13.94) | 0.168 |
| CYFRA 21-1 (ng/ml) | 3.35 (2.23-5.76) | 3.98 (2.56-6.54) | 0.067 |
| NSE (ng/ml) | 14.95 (11.99-20.31) | 15.37 (12.25-19.33) | 0.842 |
| SCC-Ag (ng/ml) | 0.84 (0.60-1.23) | 0.85 (0.59-1.20) | 0.925 |
| SUV_max_ | 11.29(4.54-17.68) | 14.63(6.71-19.02) | 0.028 |
| EGFR |  |  | 0.534 |
| Wild-type | 161 (39.85%) | 41 (36.94%) |  |
| Mutant | 243 (60.15%) | 70 (63.06%) |  |

Note: Mean (SD) / Median (Q1-Q3) / N (%) . EGFR, epidermal growth factor receptor; CEA, carcinoembryonic antigen; CYFRA 21-1, cytokeratin 19 fragment; NSE, neuron-specific enolase; SCC-Ag, squamous cell carcinoma associated antigen; SUV_max_, maximum standardized uptake value.

**Table S3: Radiomics features used by the three modality models**

| **Model** | **Radiomics features** |
| --- | --- |
| **CT_RS** | original_firstorder_Kurtosis  original_firstorder_Median  original_firstorder_Skewness  log-sigma-1-0-mm-3D_firstorder_Energy  log-sigma-4-0-mm-3D_gldm_DependenceVariance  wavelet-LHL_glrlm_LongRunLowGrayLevelEmphasis  wavelet-HLL_firstorder_Energy  wavelet-HHL_firstorder_Kurtosis |
| **PET_RS** | original_shape_Maximum2DDiameterColumn  log-sigma-0-5-mm-3D_gldm_LargeDependenceLowGrayLevelEmphasis  log-sigma-2-5-mm-3D_glszm_ZoneEntropy  log-sigma-5-0-mm-3D_ngtdm_Coarseness |
| **PET/CT_RS** | original_firstorder_Median  wavelet-HHL_firstorder_Kurtosis  original_shape_Maximum2DDiameterColumn  log-sigma-2-5-mm-3D_glszm_ZoneEntropy |

Note: RS, Rad-score.

**Table S4: Clinical characteristics and the EGFR mutation rate of patients in clinical stage Ⅰ-Ⅱ group and Ⅲ-Ⅳ group**

| Clinical stage | **Ⅰ-Ⅱ** | **Ⅲ-Ⅳ** | P-value |
| --- | --- | --- | --- |
| N | 233 | 282 |  |
| Age (years) | 64.42 (9.12) | 63.66 (9.23) | 0.360 |
| Gender |  |  | 0.002 |
| Female | 137 (58.8%) | 127 (45%) |  |
| Male | 96 (41.2%) | 155 (55%) |  |
| Smoking history | 60 (25.8%) | 115 (40.8%) | <0.001 |
| Nodule type |  |  | <0.001 |
| Solidity | 90 (38.6%) | 258 (91.5%) |  |
| Sub-solidity | 143 (61.4%) | 24 (8.5%) |  |
| Nodule location |  |  | 0.242 |
| Top right | 78 (33.5%) | 81 (28.7%) |  |
| Middle right | 14 (6.0%) | 18 (6.4%) |  |
| Lower right | 44 (18.9%) | 66 (23.4%) |  |
| Top left | 68 (29.2%) | 68 (24.1%) |  |
| Lower left | 29 (12.4%) | 49 (17.4%) |  |
| Tumor long axis | 22.60 (17.40 – 28.90) | 37.85 (26.47 – 50.55) | <0.001 |
| Tumor short axis | 16.40 (12.30 – 20.60) | 28.05 (19.15 – 36.22) | <0.001 |
| CEA | 2.54 (1.29 – 4.71) | 9.38 (3.33 – 43.26) | <0.001 |
| CYFRA 21-1 | 2.42 (1.86 – 3.52) | 4.96 (3.14 – 7.97) | <0.001 |
| NSE | 13.07 (10.73 – 16.73) | 17.16 (13.67 – 21.94) | <0.001 |
| SCCag | 0.79 (0.54 – 1.13) | 0.90 (0.65 – 1.38) | <0.001 |
| SUVmax | 4.61 (2.40 – 9.98) | 16.29 (12.41 – 21.37) | <0.001 |
| EGFR |  |  | 0.005 |
| Wild-type | 76 (32.6%) | 126 (44.7%) |  |
| Mutant | 157 (67.4%) | 156 (55.3%) |  |

Note: Mean (SD) / Median (Q1-Q3) / N (%) . EGFR, epidermal growth factor receptor; CEA, carcinoembryonic antigen; CYFRA 21-1, cytokeratin 19 fragment; NSE, neuron-specific enolase; SCC-Ag, squamous cell carcinoma associated antigen; SUV_max_, maximum standardized uptake value.


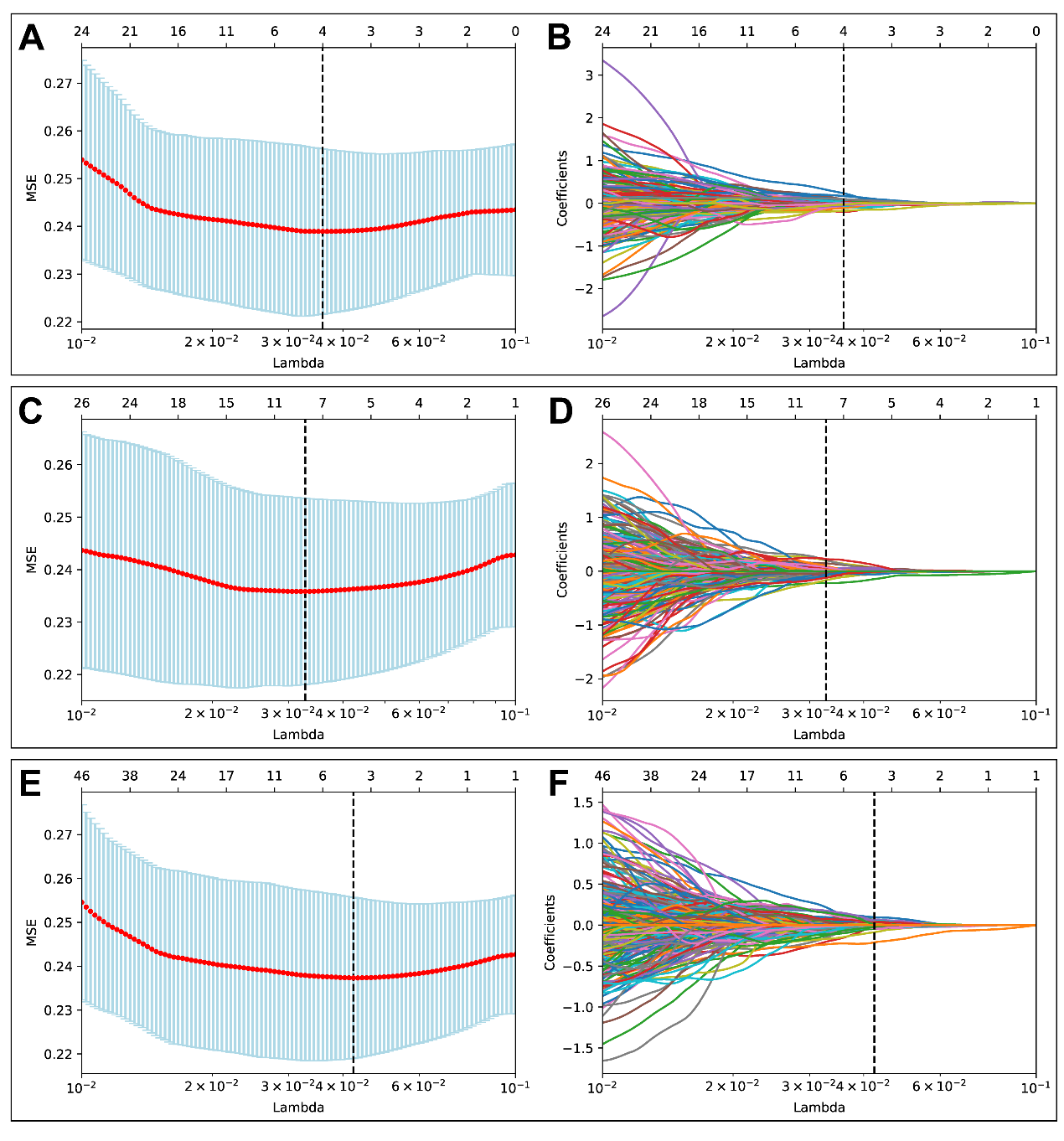


**Figure S1:** The LASSO algorithm and 5-fold cross-validation were used to extract the optimal subset of radiomic features. (A,C,E) The best feature was selected based on the AUC value. The black vertical line defines the best value of λ, and the model provides the best fit of the data. (B, D, E) The LASSO coefficient profiles of PET, CT, PET/CT radiomic features. LASSO, least absolute shrinkage and selection operator; AUC, area under the curve.


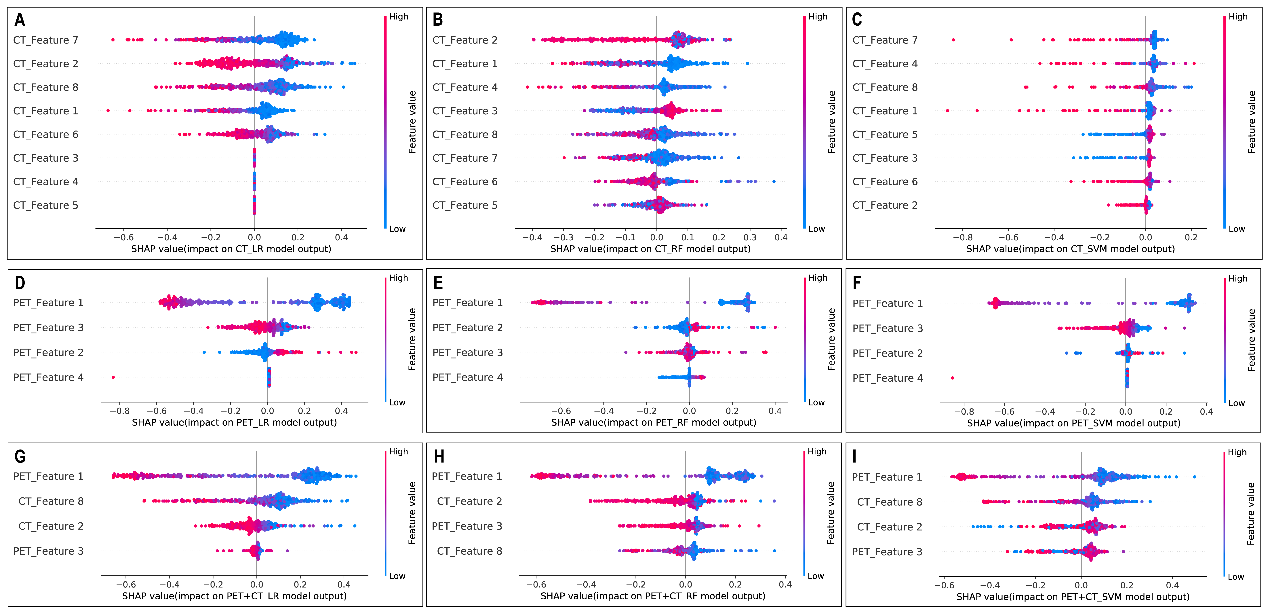


**Figure** **S2:** SHAP value graph of CT (A-C) ,PET (D-F) and PET/CT (G-I) radiomics models.

CT_Feature 1：original_firstorder_Kurtosis；

CT_Feature 2：original_firstorder_Median；

CT_Feature 3：original_firstorder_Skewness；

CT_Feature 4：log-sigma-1-0-mm-3D_firstorder_Energy；

CT_Feature 5：log-sigma-4-0-mm-3D_gldm_DependenceVariance；

CT_Feature 6：wavelet-LHL_glrlm_LongRunLowGrayLevelEmphasis；

CT_Feature 7：wavelet-HLL_firstorder_Energy；

CT_Feature 8：wavelet-HHL_firstorder_Kurtosis；

PET_Feature 1：original_shape_Maximum2DDiameterColumn；

PET_Feature 2：log-sigma-0-5-mm-3D_gldm_LargeDependenceLowGrayLevelEmphasis；

PET_Feature 3：log-sigma-2-5-mm-3D_glszm_ZoneEntropy；

PET_Feature 4：log-sigma-5-0-mm-3D_ngtdm_Coarseness


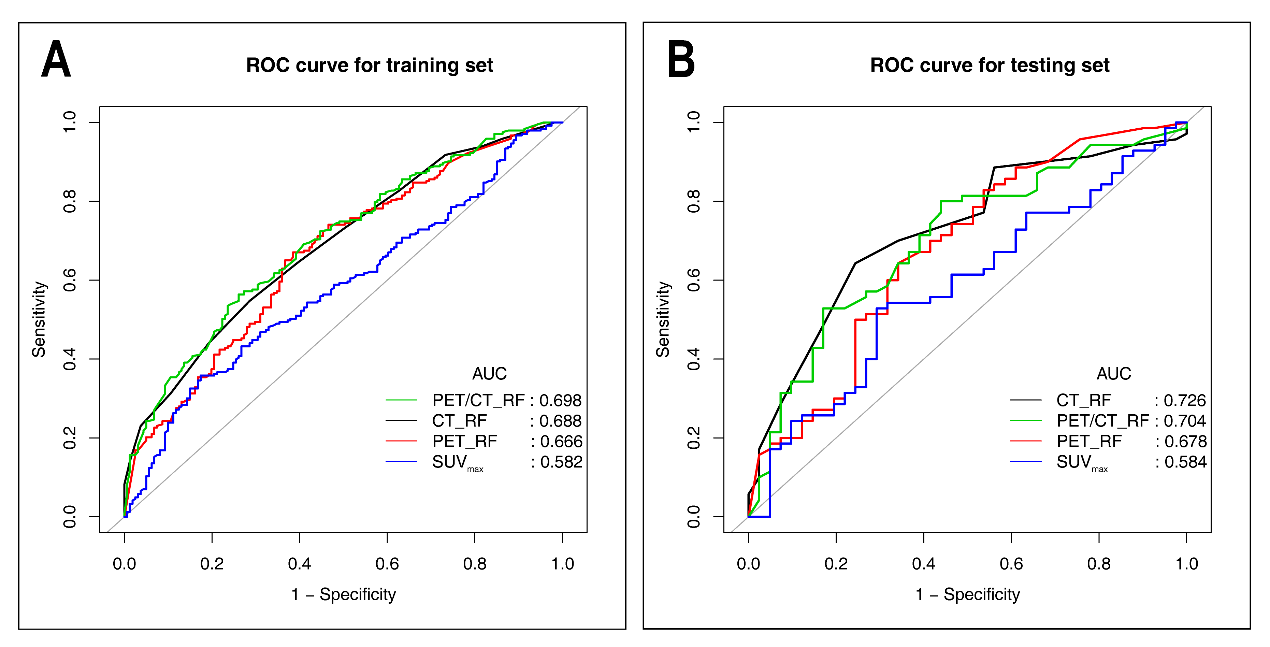


**Figure S3:** The ROC curve of the three best radiomics models and SUV_max_ for identifying EGFR mutation status in training set (A) and testing set (B). AUC, area under the curve; ROC, receiver operating characteristic; RF, random forest.
